# Bis(2,2′-bipyridine-κ^2^ *N*,*N*′)(nitrato-κ*O*)copper(II) perchlorate

**DOI:** 10.1107/S1600536811002571

**Published:** 2011-02-05

**Authors:** Yu Zhu, Yun-Long Wu, Chun-Xia Huang, Ji-Min Xie

**Affiliations:** a School of Chemistry and Chemical Engineering, Jiangsu University, Zhenjiang 212013, People’s Republic of China

## Abstract

In the title compound, [Cu(NO_3_)(C_10_H_8_N_2_)_2_]ClO_4_, the five-coordinated Cu^II^ atom has a distorted square-pyramidal CuN_4_O environment; the O atom is in the axial position whereas the N atoms from two bipyridine (bipy) ligands are in the equatorial plane. In the crystal, mol­ecules are assembled by C—H⋯O hydrogen bonding and π–π inter­actions between bipy groups [centroid–centroid distances = 3.7686 (16) and 3.7002 (16) Å] into a three-dimensional network. The nitrite anion is equally disordered over two sets of sites.

## Related literature

For the applications of complexes with bipyridine and its derivatives in catalysis and visible-light-driven water oxidation, see: Morrow & Trogler (1989 ▶) and Duan *et al.* (2010 ▶), respectively. 
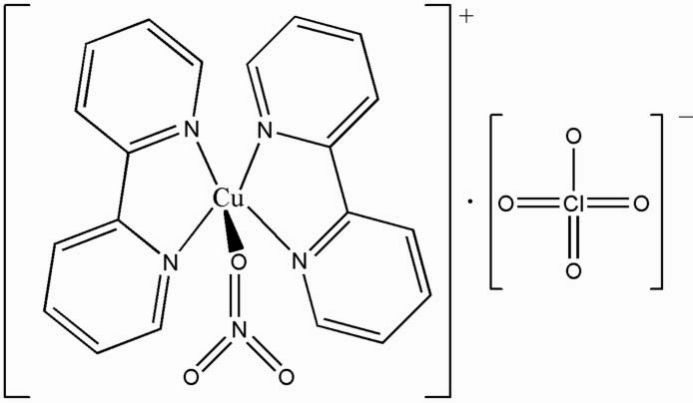


## Experimental

### 

#### Crystal data

[Cu(NO_3_)(C_10_H_8_N_2_)_2_]ClO_4_
*M* *_r_* = 537.38
Triclinic, 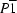

*a* = 7.5882 (15) Å
*b* = 10.473 (2) Å
*c* = 14.041 (3) Å
α = 76.15 (3)°
β = 81.46 (4)°
γ = 78.86 (3)°
*V* = 1056.9 (4) Å^3^
*Z* = 2
Mo *K*α radiation
μ = 1.22 mm^−1^
*T* = 295 K
0.24 × 0.20 × 0.18 mm

#### Data collection

Rigaku Saturn 724 diffractometer
Absorption correction: multi-scan (*CrystalClear*; Rigaku, 2007 ▶) *T*_min_ = 0.747, *T*_max_ = 0.803
10121 measured reflections
4037 independent reflections
3579 reflections with *I* > 2σ(*I*)
*R*_int_ = 0.019

#### Refinement

*R*[*F* ^2^ > 2σ(*F* ^2^)] = 0.034
*wR*(*F* ^2^) = 0.088
*S* = 1.05
4037 reflections
351 parameters
H-atom parameters constrained
Δρ_max_ = 0.31 e Å^−3^
Δρ_min_ = −0.48 e Å^−3^

### 

Data collection: *CrystalClear* (Rigaku, 2007 ▶); cell refinement: *CrystalClear*; data reduction: *CrystalClear*; program(s) used to solve structure: *SHELXS97* (Sheldrick, 2008 ▶); program(s) used to refine structure: *SHELXL97* (Sheldrick, 2008 ▶); molecular graphics: *SHELXTL* (Sheldrick, 2008 ▶); software used to prepare material for publication: *SHELXL97*.

## Supplementary Material

Crystal structure: contains datablocks I, global. DOI: 10.1107/S1600536811002571/kp2303sup1.cif

Structure factors: contains datablocks I. DOI: 10.1107/S1600536811002571/kp2303Isup2.hkl

Additional supplementary materials:  crystallographic information; 3D view; checkCIF report

## Figures and Tables

**Table 1:** Selected bond lengths (Å)

Cu1—N1	1.986 (2)
Cu1—N4	1.9890 (19)
Cu1—N2	2.0426 (19)
Cu1—N3	2.0534 (18)
Cu1—O3	2.38 (4)

**Table 2:** Hydrogen-bond geometry (Å, °)

*D*—H⋯*A*	*D*—H	H⋯*A*	*D*⋯*A*	*D*—H⋯*A*
C3—H3⋯O5^i^	0.93	2.58	3.276 (3)	132
C7—H7⋯O4^ii^	0.93	2.53	3.322 (4)	144
C13—H13⋯O7^iii^	0.93	2.43	3.249 (3)	147

Symmetry codes: (i) 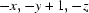; (ii) 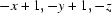; (iii) 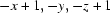.

**Fig. 1.**
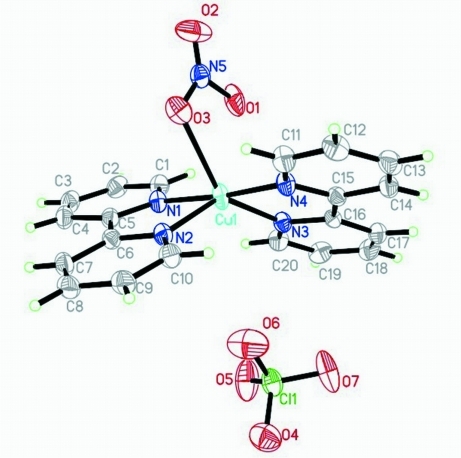
View of the title complex showing the labeling of the non-H atoms and 30% probability displacement ellipsoids. H atoms are shown as spheres of arbitrary radii. Disorders of the coordinated [NO3]- and uncoordinated [ClO4]-anions are not shown.

**Fig. 2.**
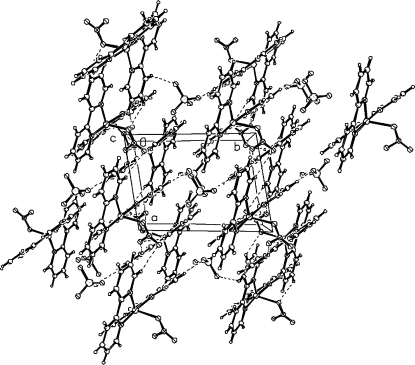
Three dimensional architecture constructed by intermolecular C—H···O hydrogen bonding (dashed lines) and π-π interactions.

## supplementary crystallographic information

### Comment

Complexes with bipyridine and its derivatives have been extensively studied because of their potential applications in catalysis (Morrow & Trogler, 1989) and visible light driven water oxidation (Duan *et al.*, 2010). Herein we report the synthesis and structure of the title copper complex with 2, 2'-bipyridine.

The structure of the title complex (Fig. 1)consists of a discrete cation [Cu(bipy)_2_(NO_3_)]^+^ and an uncoordinated ClO_4_^-^ anion which is in disorder. The Cu(II) atom is five coordinated by four nitrogen atoms from two bipy ligands and one oxygen atom from one nitrate anion, exhibiting a distorted square pyramidal coordination with the oxygen atom in the axial position (Fig. 1 and Table 1). The uncoordinated perchlorate anion displays the expected tetrahedral geometry. There are weak intermolecular C—H···O hydrogen bonds in the crystal structure(C3—H3···O5^i^, C7—H7···O4^ii^ and C13—H13···O7^iii^; Table 2). Crystal packing is stabilised by the C—H···O hydrogen bonds and π-π interactions between two parallel bipy rings [centroid (N1, C1—C5)···centroid (N1, C1—C5)^iv^ = 3.77 Å; centroid (N2, C6—C10) ···centroid (N2, C6—C10)^v^= 3.70 Å; centroid (N3, C16—C20)···centroid (N4, C11—C15)^vi^ = 3.75 Å; symmetry codes: (iv)-*x*, 1 - *y*, -*z*; (v) 1 - *x*, -*y*, -*z*; (vi) -*x*, -*y*, 1 - *z*)](Fig. 2).

### Experimental

2, 2'-bipyridine (31.3 mg, 0.2 mmol), Cu(NO_3_)_2_.3H_2_O(42 mg, 0.2 mmol), NaClO_4_ (28 mg, 0.2 mmol), acetone (10 mL) and methanol (6 mL) were stirred for 8 h at 313 K. The solution was then filtered, evaporated in the air and prismatic blue crystals were formed after 2 days (yieled 78%).

### Refinement

All the H atoms were placed in calculated positions and refined using a riding model, with *U*_iso_ (H) =1.2*U*_eq_(C, N) and C–H =0.93 and N–H=0.86 Å. The disorder of [NO_3_]^-^ with two locations of O1, O2, and O3 led in the refinement to 1:1 ratio in occupancy for each oxygen atom. However, a slight disorder of [ClO_4_]^-^ cannot be described geometrically.

### Crystal data

**Table:**

[Cu(NO_3_)(C_10_H_8_N_2_)_2_]ClO_4_	*Z* = 2
*M**_r_* = 537.38	*F*(000) = 546
Triclinic, *P*1	*D*_x_ = 1.689 Mg m^−^^3^
Hall symbol: -P 1	Mo *K*α radiation, λ = 0.71073 Å
*a* = 7.5882 (15) Å	Cell parameters from 4693 reflections
*b* = 10.473 (2) Å	θ = 3.1–29.0°
*c* = 14.041 (3) Å	µ = 1.22 mm^−^^1^
α = 76.15 (3)°	*T* = 295 K
β = 81.46 (4)°	Prism, blue
γ = 78.86 (3)°	0.24 × 0.20 × 0.18 mm
*V* = 1056.9 (4) Å^3^	

### Data collection

**Table:**

Rigaku Saturn 724 diffractometer	4037 independent reflections
Radiation source: fine-focus sealed tube	3579 reflections with *I* > 2σ(*I*)
graphite	*R*_int_ = 0.019
ω scans	θ_max_ = 26.0°, θ_min_ = 3.1°
Absorption correction: multi-scan (*CrystalClear*; Rigaku, 2007)	*h* = −8→9
*T*_min_ = 0.747, *T*_max_ = 0.803	*k* = −12→12
10121 measured reflections	*l* = −17→17

### Refinement

**Table:**

Refinement on *F*^2^	Primary atom site location: structure-invariant direct methods
Least-squares matrix: full	Secondary atom site location: difference Fourier map
*R*[*F*^2^ > 2σ(*F*^2^)] = 0.034	Hydrogen site location: inferred from neighbouring sites
*wR*(*F*^2^) = 0.088	H-atom parameters constrained
*S* = 1.05	*w* = 1/[σ^2^(*F*_o_^2^) + (0.0453*P*)^2^ + 0.3686*P*] where *P* = (*F*_o_^2^ + 2*F*_c_^2^)/3
4037 reflections	(Δ/σ)_max_ < 0.001
351 parameters	Δρ_max_ = 0.31 e Å^−^^3^
0 restraints	Δρ_min_ = −0.48 e Å^−^^3^

### Special details

**Table:**

Geometry. All e.s.d.'s (except the e.s.d. in the dihedral angle between two l.s. planes) are estimated using the full covariance matrix. The cell e.s.d.'s are taken into account individually in the estimation of e.s.d.'s in distances, angles and torsion angles; correlations between e.s.d.'s in cell parameters are only used when they are defined by crystal symmetry. An approximate (isotropic) treatment of cell e.s.d.'s is used for estimating e.s.d.'s involving l.s. planes.
Refinement. Refinement of *F*^2^ against ALL reflections. The weighted *R*-factor *wR* and goodness of fit *S* are based on *F*^2^, conventional *R*-factors *R* are based on *F*, with *F* set to zero for negative *F*^2^. The threshold expression of *F*^2^ > σ(*F*^2^) is used only for calculating *R*-factors(gt) *etc*. and is not relevant to the choice of reflections for refinement. *R*-factors based on *F*^2^ are statistically about twice as large as those based on *F*, and *R*- factors based on ALL data will be even larger.

### Fractional atomic coordinates and isotropic or equivalent isotropic displacement parameters (Å^2^)

**Table:**

	*x*	*y*	*z*	*U*_iso_*/*U*_eq_	Occ. (<1)
Cu1	0.13510 (4)	0.14256 (3)	0.229930 (18)	0.04365 (11)	
N1	−0.0008 (3)	0.26435 (18)	0.12391 (13)	0.0413 (4)	
C1	−0.1751 (3)	0.3189 (2)	0.13465 (18)	0.0460 (5)	
H1	−0.2386	0.3047	0.1972	0.062 (8)*	
O1	−0.144 (6)	0.025 (3)	0.2791 (18)	0.072 (5)	0.42 (7)
O1'	−0.092 (3)	0.0253 (18)	0.2643 (12)	0.054 (3)	0.58 (7)
Cl1	0.40225 (9)	0.48718 (7)	0.29287 (4)	0.05741 (18)	
N2	0.3386 (2)	0.15087 (18)	0.11680 (13)	0.0405 (4)	
C2	−0.2638 (4)	0.3950 (2)	0.05677 (19)	0.0526 (6)	
H2	−0.3832	0.4358	0.0670	0.074 (9)*	
O2	−0.165 (6)	−0.127 (4)	0.212 (3)	0.093 (7)	0.42 (7)
O2'	−0.171 (4)	−0.151 (2)	0.2322 (14)	0.071 (3)	0.58 (7)
N3	0.0518 (2)	0.23533 (17)	0.34604 (13)	0.0377 (4)	
C3	−0.1733 (4)	0.4104 (3)	−0.03679 (19)	0.0561 (7)	
H3	−0.2314	0.4605	−0.0909	0.063 (8)*	
O3	0.070 (2)	−0.033 (3)	0.1678 (19)	0.063 (5)	0.42 (7)
O3'	0.047 (3)	−0.0583 (17)	0.1459 (15)	0.082 (3)	0.58 (7)
N4	0.2614 (2)	0.00723 (18)	0.33384 (13)	0.0412 (4)	
C4	0.0043 (4)	0.3504 (2)	−0.04923 (17)	0.0514 (6)	
H4	0.0664	0.3571	−0.1121	0.070 (9)*	
O4	0.5696 (3)	0.5366 (3)	0.26537 (19)	0.0918 (7)	
N5	−0.0741 (3)	−0.0584 (2)	0.21550 (15)	0.0465 (5)	
C5	0.0898 (3)	0.2800 (2)	0.03288 (15)	0.0406 (5)	
O5	0.2613 (4)	0.5915 (3)	0.25784 (17)	0.1054 (9)	
C6	0.2824 (3)	0.2197 (2)	0.02926 (15)	0.0398 (5)	
O6	0.4111 (4)	0.3772 (3)	0.2508 (2)	0.1198 (11)	
C7	0.4017 (4)	0.2356 (2)	−0.05599 (17)	0.0496 (6)	
H7	0.3614	0.2841	−0.1156	0.055 (7)*	
O7	0.3702 (4)	0.4516 (2)	0.39655 (15)	0.0995 (9)	
C8	0.5801 (4)	0.1788 (3)	−0.05135 (19)	0.0534 (6)	
H8	0.6618	0.1882	−0.1079	0.054 (7)*	
C9	0.6372 (3)	0.1078 (3)	0.03760 (19)	0.0516 (6)	
H9	0.7572	0.0679	0.0420	0.075 (9)*	
C10	0.5128 (3)	0.0971 (2)	0.11998 (18)	0.0476 (5)	
H10	0.5518	0.0505	0.1804	0.053 (7)*	
C11	0.3604 (3)	−0.1094 (2)	0.32214 (18)	0.0510 (6)	
H11	0.3782	−0.1292	0.2599	0.062 (8)*	
C12	0.4372 (3)	−0.2012 (2)	0.3986 (2)	0.0541 (6)	
H12	0.5064	−0.2812	0.3883	0.065 (8)*	
C13	0.4095 (3)	−0.1719 (2)	0.4905 (2)	0.0532 (6)	
H13	0.4616	−0.2316	0.5432	0.059 (8)*	
C14	0.3040 (3)	−0.0538 (2)	0.50390 (17)	0.0451 (5)	
H14	0.2812	−0.0342	0.5662	0.052 (7)*	
C15	0.2321 (3)	0.0353 (2)	0.42427 (15)	0.0363 (5)	
C16	0.1165 (3)	0.1643 (2)	0.43096 (15)	0.0356 (4)	
C17	0.0741 (3)	0.2098 (2)	0.51762 (16)	0.0452 (5)	
H17	0.1225	0.1607	0.5750	0.055 (7)*	
C18	−0.0408 (4)	0.3290 (3)	0.51792 (19)	0.0541 (6)	
H18	−0.0735	0.3599	0.5759	0.061 (8)*	
C19	−0.1059 (3)	0.4013 (3)	0.4320 (2)	0.0541 (6)	
H19	−0.1838	0.4819	0.4308	0.062 (8)*	
C20	−0.0546 (3)	0.3528 (2)	0.34734 (18)	0.0478 (6)	
H20	−0.0957	0.4039	0.2886	0.051 (7)*	

### Atomic displacement parameters (Å^2^)

**Table:**

	*U*^11^	*U*^22^	*U*^33^	*U*^12^	*U*^13^	*U*^23^
Cu1	0.04732 (18)	0.04701 (18)	0.02684 (15)	0.00769 (13)	0.00054 (11)	−0.00424 (11)
N1	0.0475 (10)	0.0413 (10)	0.0313 (9)	−0.0004 (8)	−0.0030 (8)	−0.0065 (8)
C1	0.0471 (13)	0.0464 (13)	0.0430 (13)	−0.0012 (11)	−0.0041 (10)	−0.0124 (10)
O1	0.103 (12)	0.060 (5)	0.036 (5)	0.009 (7)	0.021 (7)	−0.012 (4)
O1'	0.068 (5)	0.060 (3)	0.031 (4)	−0.014 (4)	0.014 (3)	−0.012 (3)
Cl1	0.0584 (4)	0.0596 (4)	0.0441 (3)	0.0051 (3)	0.0029 (3)	−0.0088 (3)
N2	0.0449 (10)	0.0414 (10)	0.0325 (9)	−0.0048 (8)	0.0011 (8)	−0.0076 (8)
C2	0.0539 (15)	0.0484 (14)	0.0573 (15)	0.0009 (12)	−0.0205 (12)	−0.0135 (12)
O2	0.088 (11)	0.054 (8)	0.136 (17)	−0.029 (5)	−0.030 (12)	0.004 (9)
O2'	0.079 (5)	0.065 (8)	0.074 (5)	−0.037 (6)	−0.002 (4)	−0.008 (5)
N3	0.0402 (9)	0.0369 (9)	0.0334 (9)	−0.0021 (8)	−0.0014 (7)	−0.0076 (7)
C3	0.0705 (17)	0.0509 (15)	0.0477 (14)	−0.0035 (13)	−0.0271 (13)	−0.0050 (11)
O3	0.047 (4)	0.065 (7)	0.064 (7)	−0.001 (4)	0.004 (4)	−0.006 (4)
O3'	0.065 (4)	0.099 (5)	0.084 (5)	−0.022 (4)	0.030 (4)	−0.042 (5)
N4	0.0443 (10)	0.0405 (10)	0.0328 (9)	0.0020 (8)	−0.0006 (8)	−0.0059 (8)
C4	0.0746 (17)	0.0465 (13)	0.0325 (12)	−0.0088 (12)	−0.0098 (12)	−0.0059 (10)
O4	0.0823 (16)	0.0942 (17)	0.0956 (17)	−0.0216 (13)	0.0108 (13)	−0.0207 (14)
N5	0.0438 (12)	0.0518 (13)	0.0397 (11)	−0.0028 (11)	−0.0084 (9)	−0.0034 (10)
C5	0.0555 (13)	0.0339 (11)	0.0321 (11)	−0.0064 (10)	−0.0045 (10)	−0.0076 (9)
O5	0.1019 (18)	0.125 (2)	0.0643 (14)	0.0473 (16)	−0.0261 (13)	−0.0138 (14)
C6	0.0537 (13)	0.0335 (11)	0.0323 (11)	−0.0077 (10)	0.0013 (10)	−0.0106 (9)
O6	0.1064 (19)	0.111 (2)	0.157 (3)	−0.0352 (17)	0.0496 (19)	−0.084 (2)
C7	0.0662 (16)	0.0484 (13)	0.0329 (12)	−0.0125 (12)	0.0050 (11)	−0.0104 (10)
O7	0.1140 (19)	0.0939 (16)	0.0472 (11)	0.0367 (14)	0.0124 (12)	0.0126 (11)
C8	0.0591 (15)	0.0578 (15)	0.0456 (14)	−0.0197 (13)	0.0178 (12)	−0.0221 (12)
C9	0.0467 (14)	0.0554 (15)	0.0536 (15)	−0.0114 (12)	0.0077 (11)	−0.0192 (12)
C10	0.0461 (13)	0.0499 (13)	0.0447 (13)	−0.0046 (11)	−0.0009 (11)	−0.0112 (11)
C11	0.0565 (14)	0.0455 (13)	0.0459 (14)	0.0046 (11)	−0.0013 (11)	−0.0128 (11)
C12	0.0505 (14)	0.0396 (13)	0.0657 (17)	0.0041 (11)	−0.0075 (12)	−0.0071 (11)
C13	0.0502 (14)	0.0467 (14)	0.0559 (15)	−0.0061 (11)	−0.0171 (12)	0.0076 (11)
C14	0.0453 (13)	0.0498 (13)	0.0380 (12)	−0.0101 (11)	−0.0084 (10)	−0.0013 (10)
C15	0.0344 (10)	0.0401 (11)	0.0330 (11)	−0.0086 (9)	−0.0013 (9)	−0.0047 (9)
C16	0.0357 (11)	0.0393 (11)	0.0310 (10)	−0.0102 (9)	0.0017 (8)	−0.0060 (8)
C17	0.0505 (13)	0.0527 (13)	0.0337 (12)	−0.0123 (11)	0.0009 (10)	−0.0121 (10)
C18	0.0565 (15)	0.0630 (16)	0.0495 (14)	−0.0128 (13)	0.0057 (12)	−0.0298 (13)
C19	0.0524 (14)	0.0462 (14)	0.0661 (17)	0.0012 (12)	−0.0043 (12)	−0.0254 (12)
C20	0.0515 (13)	0.0412 (12)	0.0484 (13)	0.0005 (11)	−0.0066 (11)	−0.0106 (10)

### Geometric parameters (Å, °)

**Table:**

Cu1—N1	1.986 (2)	N4—C11	1.338 (3)
Cu1—N4	1.9890 (19)	N4—C15	1.348 (3)
Cu1—N2	2.0426 (19)	C4—C5	1.387 (3)
Cu1—N3	2.0534 (18)	C4—H4	0.9301
Cu1—O1'	2.233 (15)	C5—C6	1.474 (3)
Cu1—O3	2.38 (4)	C6—C7	1.386 (3)
Cu1—O1	2.57 (5)	C7—C8	1.374 (4)
Cu1—O3'	2.87 (3)	C7—H7	0.9301
N1—C1	1.338 (3)	C8—C9	1.375 (4)
N1—C5	1.347 (3)	C8—H8	0.9300
C1—C2	1.370 (3)	C9—C10	1.377 (3)
C1—H1	0.9299	C9—H9	0.9300
O1—N5	1.38 (2)	C10—H10	0.9299
O1'—N5	1.213 (19)	C11—C12	1.375 (3)
Cl1—O6	1.402 (3)	C11—H11	0.9300
Cl1—O7	1.410 (2)	C12—C13	1.374 (4)
Cl1—O5	1.424 (2)	C12—H12	0.9300
Cl1—O4	1.429 (2)	C13—C14	1.375 (4)
N2—C10	1.336 (3)	C13—H13	0.9300
N2—C6	1.351 (3)	C14—C15	1.380 (3)
C2—C3	1.377 (4)	C14—H14	0.9300
C2—H2	0.9301	C15—C16	1.477 (3)
O2—N5	1.10 (4)	C16—C17	1.383 (3)
O2'—N5	1.28 (2)	C17—C18	1.378 (4)
N3—C20	1.337 (3)	C17—H17	0.9300
N3—C16	1.350 (3)	C18—C19	1.367 (4)
C3—C4	1.378 (4)	C18—H18	0.9300
C3—H3	0.9299	C19—C20	1.378 (3)
O3—N5	1.231 (16)	C19—H19	0.9300
O3'—N5	1.238 (13)	C20—H20	0.9300
			
N1—Cu1—N4	174.76 (8)	C5—C4—H4	120.3
N1—Cu1—N2	81.03 (8)	O2—N5—O1'	130.3 (17)
N4—Cu1—N2	99.78 (8)	O2—N5—O3	133.1 (18)
N1—Cu1—N3	101.94 (8)	O1'—N5—O3	96.5 (13)
N4—Cu1—N3	81.12 (7)	O2—N5—O3'	109.6 (16)
N2—Cu1—N3	135.94 (7)	O1'—N5—O3'	119.0 (9)
N1—Cu1—O1'	87.4 (4)	O1'—N5—O2'	124.5 (11)
N4—Cu1—O1'	88.2 (4)	O3—N5—O2'	138 (2)
N2—Cu1—O1'	130.0 (5)	O3'—N5—O2'	116.5 (15)
N3—Cu1—O1'	94.0 (5)	O2—N5—O1	113 (2)
N1—Cu1—O3	85.8 (4)	O3—N5—O1	113.3 (9)
N4—Cu1—O3	89.1 (4)	O3'—N5—O1	135.0 (10)
N2—Cu1—O3	84.1 (4)	O2'—N5—O1	108 (2)
N3—Cu1—O3	139.8 (4)	N1—C5—C4	120.9 (2)
O1'—Cu1—O3	46.5 (6)	N1—C5—C6	114.93 (19)
N1—Cu1—O1	86.4 (5)	C4—C5—C6	124.1 (2)
N4—Cu1—O1	89.5 (5)	N2—C6—C7	121.3 (2)
N2—Cu1—O1	135.3 (5)	N2—C6—C5	115.16 (18)
N3—Cu1—O1	88.6 (5)	C7—C6—C5	123.5 (2)
O1'—Cu1—O1	5.8 (7)	C8—C7—C6	119.2 (2)
O3—Cu1—O1	52.1 (6)	C8—C7—H7	120.4
N1—Cu1—O3'	82.6 (3)	C6—C7—H7	120.4
N4—Cu1—O3'	92.3 (3)	C7—C8—C9	119.5 (2)
N2—Cu1—O3'	83.3 (2)	C7—C8—H8	120.3
N3—Cu1—O3'	140.7 (3)	C9—C8—H8	120.2
O1'—Cu1—O3'	46.9 (6)	C8—C9—C10	118.6 (2)
O3—Cu1—O3'	3.3 (5)	C8—C9—H9	120.7
O1—Cu1—O3'	52.5 (6)	C10—C9—H9	120.7
C1—N1—C5	119.1 (2)	N2—C10—C9	122.8 (2)
C1—N1—Cu1	125.44 (16)	N2—C10—H10	118.6
C5—N1—Cu1	115.08 (15)	C9—C10—H10	118.6
N1—C1—C2	122.4 (2)	N4—C11—C12	122.5 (2)
N1—C1—H1	118.9	N4—C11—H11	118.8
C2—C1—H1	118.8	C12—C11—H11	118.8
N5—O1—Cu1	90 (2)	C13—C12—C11	118.5 (2)
N5—O1'—Cu1	113.1 (13)	C13—C12—H12	120.8
O6—Cl1—O7	111.08 (19)	C11—C12—H12	120.7
O6—Cl1—O5	111.0 (2)	C12—C13—C14	119.5 (2)
O7—Cl1—O5	108.19 (14)	C12—C13—H13	120.2
O6—Cl1—O4	108.65 (16)	C14—C13—H13	120.3
O7—Cl1—O4	109.62 (17)	C13—C14—C15	119.5 (2)
O5—Cl1—O4	108.20 (18)	C13—C14—H14	120.2
C10—N2—C6	118.6 (2)	C15—C14—H14	120.2
C10—N2—Cu1	128.24 (16)	N4—C15—C14	120.9 (2)
C6—N2—Cu1	113.12 (15)	N4—C15—C16	115.32 (18)
C1—C2—C3	119.0 (2)	C14—C15—C16	123.7 (2)
C1—C2—H2	120.5	N3—C16—C17	121.7 (2)
C3—C2—H2	120.5	N3—C16—C15	115.19 (18)
C20—N3—C16	118.09 (19)	C17—C16—C15	123.1 (2)
C20—N3—Cu1	128.80 (16)	C18—C17—C16	119.2 (2)
C16—N3—Cu1	113.10 (14)	C18—C17—H17	120.3
C2—C3—C4	119.1 (2)	C16—C17—H17	120.5
C2—C3—H3	120.5	C19—C18—C17	119.3 (2)
C4—C3—H3	120.4	C19—C18—H18	120.4
N5—O3—Cu1	104 (2)	C17—C18—H18	120.4
N5—O3'—Cu1	80.4 (14)	C18—C19—C20	118.9 (2)
C11—N4—C15	119.03 (19)	C18—C19—H19	120.5
C11—N4—Cu1	125.60 (16)	C20—C19—H19	120.6
C15—N4—Cu1	115.23 (14)	N3—C20—C19	122.8 (2)
C3—C4—C5	119.4 (2)	N3—C20—H20	118.6
C3—C4—H4	120.3	C19—C20—H20	118.6
			
N2—Cu1—N1—C1	−179.4 (2)	O1—Cu1—N4—C11	88.5 (5)
N3—Cu1—N1—C1	−44.1 (2)	O3'—Cu1—N4—C11	36.1 (3)
O1'—Cu1—N1—C1	49.5 (6)	N2—Cu1—N4—C15	136.89 (16)
O3—Cu1—N1—C1	96.0 (4)	N3—Cu1—N4—C15	1.56 (15)
O1—Cu1—N1—C1	43.7 (5)	O1'—Cu1—N4—C15	−92.8 (6)
O3'—Cu1—N1—C1	96.3 (3)	O3—Cu1—N4—C15	−139.3 (4)
N2—Cu1—N1—C5	7.62 (15)	O1—Cu1—N4—C15	−87.1 (5)
N3—Cu1—N1—C5	142.88 (15)	O3'—Cu1—N4—C15	−139.5 (3)
O1'—Cu1—N1—C5	−123.6 (6)	C2—C3—C4—C5	−2.3 (4)
O3—Cu1—N1—C5	−77.0 (4)	Cu1—O1'—N5—O2	−176 (3)
O1—Cu1—N1—C5	−129.3 (5)	Cu1—O1'—N5—O3	−0.6 (10)
O3'—Cu1—N1—C5	−76.7 (3)	Cu1—O1'—N5—O3'	−9.5 (14)
C5—N1—C1—C2	−2.7 (3)	Cu1—O1'—N5—O2'	168.7 (15)
Cu1—N1—C1—C2	−175.45 (18)	Cu1—O1'—N5—O1	−172 (6)
N1—Cu1—O1—N5	89.4 (10)	Cu1—O3—N5—O2	176 (3)
N4—Cu1—O1—N5	−87.4 (10)	Cu1—O3—N5—O1'	0.6 (9)
N2—Cu1—O1—N5	16.0 (15)	Cu1—O3—N5—O3'	161 (3)
N3—Cu1—O1—N5	−168.6 (10)	Cu1—O3—N5—O2'	−166.3 (16)
O1'—Cu1—O1—N5	−10 (8)	Cu1—O3—N5—O1	3.4 (13)
O3—Cu1—O1—N5	1.8 (7)	Cu1—O3'—N5—O2	176 (2)
O3'—Cu1—O1—N5	5.9 (7)	Cu1—O3'—N5—O1'	6.9 (10)
N1—Cu1—O1'—N5	87.2 (10)	Cu1—O3'—N5—O3	−15 (3)
N4—Cu1—O1'—N5	−90.1 (10)	Cu1—O3'—N5—O2'	−171.4 (13)
N2—Cu1—O1'—N5	11.2 (14)	Cu1—O3'—N5—O1	14.1 (15)
N3—Cu1—O1'—N5	−171.0 (10)	Cu1—O1—N5—O2	−177 (2)
O3—Cu1—O1'—N5	0.4 (7)	Cu1—O1—N5—O1'	7(5)
O1—Cu1—O1'—N5	167 (10)	Cu1—O1—N5—O3	−3.1 (12)
O3'—Cu1—O1'—N5	4.9 (7)	Cu1—O1—N5—O3'	−15.6 (17)
N1—Cu1—N2—C10	175.1 (2)	Cu1—O1—N5—O2'	169.7 (13)
N4—Cu1—N2—C10	−10.2 (2)	C1—N1—C5—C4	−0.6 (3)
N3—Cu1—N2—C10	77.1 (2)	Cu1—N1—C5—C4	172.88 (17)
O1'—Cu1—N2—C10	−106.0 (6)	C1—N1—C5—C6	178.16 (19)
O3—Cu1—N2—C10	−98.2 (4)	Cu1—N1—C5—C6	−8.3 (2)
O1—Cu1—N2—C10	−109.4 (8)	C3—C4—C5—N1	3.1 (3)
O3'—Cu1—N2—C10	−101.4 (4)	C3—C4—C5—C6	−175.6 (2)
N1—Cu1—N2—C6	−5.48 (15)	C10—N2—C6—C7	−0.3 (3)
N4—Cu1—N2—C6	169.28 (14)	Cu1—N2—C6—C7	−179.83 (17)
N3—Cu1—N2—C6	−103.42 (16)	C10—N2—C6—C5	−177.82 (19)
O1'—Cu1—N2—C6	73.4 (6)	Cu1—N2—C6—C5	2.7 (2)
O3—Cu1—N2—C6	81.2 (4)	N1—C5—C6—N2	3.6 (3)
O1—Cu1—N2—C6	70.1 (8)	C4—C5—C6—N2	−177.6 (2)
O3'—Cu1—N2—C6	78.1 (4)	N1—C5—C6—C7	−173.8 (2)
N1—C1—C2—C3	3.4 (4)	C4—C5—C6—C7	4.9 (3)
N1—Cu1—N3—C20	−4.9 (2)	N2—C6—C7—C8	0.7 (3)
N4—Cu1—N3—C20	179.4 (2)	C5—C6—C7—C8	178.0 (2)
N2—Cu1—N3—C20	84.5 (2)	C6—C7—C8—C9	−0.2 (4)
O1'—Cu1—N3—C20	−93.1 (5)	C7—C8—C9—C10	−0.6 (4)
O3—Cu1—N3—C20	−102.7 (6)	C6—N2—C10—C9	−0.6 (3)
O1—Cu1—N3—C20	−90.9 (6)	Cu1—N2—C10—C9	178.80 (17)
O3'—Cu1—N3—C20	−97.8 (6)	C8—C9—C10—N2	1.1 (4)
N1—Cu1—N3—C16	175.01 (14)	C15—N4—C11—C12	−1.4 (4)
N4—Cu1—N3—C16	−0.67 (14)	Cu1—N4—C11—C12	−176.91 (19)
N2—Cu1—N3—C16	−95.60 (16)	N4—C11—C12—C13	0.6 (4)
O1'—Cu1—N3—C16	86.8 (5)	C11—C12—C13—C14	1.1 (4)
O3—Cu1—N3—C16	77.2 (5)	C12—C13—C14—C15	−2.0 (4)
O1—Cu1—N3—C16	89.0 (6)	C11—N4—C15—C14	0.5 (3)
O3'—Cu1—N3—C16	82.1 (6)	Cu1—N4—C15—C14	176.48 (16)
C1—C2—C3—C4	−0.8 (4)	C11—N4—C15—C16	−178.1 (2)
N1—Cu1—O3—N5	−90.8 (8)	Cu1—N4—C15—C16	−2.1 (2)
N4—Cu1—O3—N5	87.9 (8)	C13—C14—C15—N4	1.1 (3)
N2—Cu1—O3—N5	−172.2 (8)	C13—C14—C15—C16	179.6 (2)
N3—Cu1—O3—N5	12.8 (12)	C20—N3—C16—C17	0.5 (3)
O1'—Cu1—O3—N5	−0.4 (7)	Cu1—N3—C16—C17	−179.47 (16)
O1—Cu1—O3—N5	−2.1 (8)	C20—N3—C16—C15	179.68 (19)
O3'—Cu1—O3—N5	−96 (11)	Cu1—N3—C16—C15	−0.3 (2)
N1—Cu1—O3'—N5	−97.8 (7)	N4—C15—C16—N3	1.6 (3)
N4—Cu1—O3'—N5	80.8 (7)	C14—C15—C16—N3	−177.00 (19)
N2—Cu1—O3'—N5	−179.6 (8)	N4—C15—C16—C17	−179.2 (2)
N3—Cu1—O3'—N5	2.0 (11)	C14—C15—C16—C17	2.2 (3)
O1'—Cu1—O3'—N5	−4.5 (7)	N3—C16—C17—C18	1.7 (3)
O3—Cu1—O3'—N5	77 (11)	C15—C16—C17—C18	−177.4 (2)
O1—Cu1—O3'—N5	−6.7 (8)	C16—C17—C18—C19	−1.9 (4)
N2—Cu1—N4—C11	−47.5 (2)	C17—C18—C19—C20	−0.1 (4)
N3—Cu1—N4—C11	177.2 (2)	C16—N3—C20—C19	−2.6 (3)
O1'—Cu1—N4—C11	82.8 (6)	Cu1—N3—C20—C19	177.36 (18)
O3—Cu1—N4—C11	36.4 (4)	C18—C19—C20—N3	2.4 (4)

### Hydrogen-bond geometry (Å, °)

**Table:**

*D*—H···*A*	*D*—H	H···*A*	*D*···*A*	*D*—H···*A*
C3—H3···O5^i^	0.93	2.58	3.276 (3)	132
C7—H7···O4^ii^	0.93	2.53	3.322 (4)	144
C13—H13···O7^iii^	0.93	2.43	3.249 (3)	147

Symmetry codes: (i) −*x*, −*y*+1, −*z*; (ii) −*x*+1, −*y*+1, −*z*; (iii) −*x*+1, −*y*, −*z*+1.
